# Boosting NIR Laser Marking Efficiency of a Transparent Epoxy Using a Layered Double Hydroxide

**DOI:** 10.1021/acsapm.4c01815

**Published:** 2024-07-05

**Authors:** Chunping Chen, Junxin Wang, Alexander Evans, Dermot O’Hare

**Affiliations:** Chemistry Research Laboratory, Department of Chemistry, University of Oxford, Oxford OX1 3TA, U.K.

**Keywords:** NIR laser marking, layered double hydroxides, transparent polymer, fast speed marking, carbonization

## Abstract

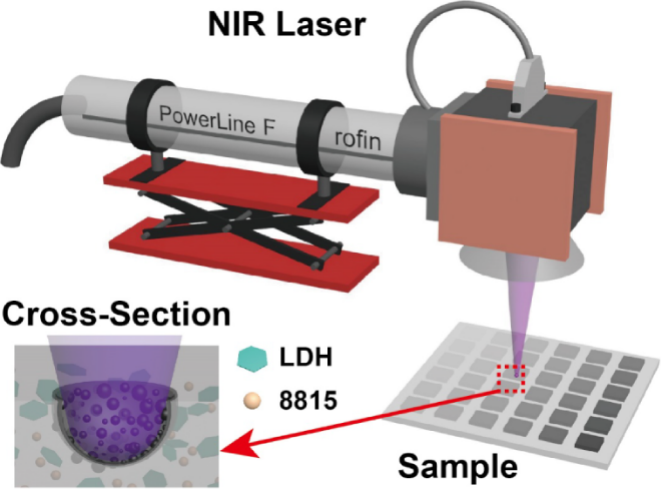

Efficient near-infrared (NIR) laser marking on transparent polymers like polypropylene, epoxy, and polyethylene has posed a big challenge due to their lack of absorption in the NIR. Currently, inorganic additives are used to improve NIR laser marking efficiency, but they come with issues such as toxicity, high loading requirement, adverse effects on color/opaqueness, and the need for low laser head speeds. Herein, we report a new strategy of incorporating a food-grade, Mg_2_Al-CO_3_ LDH as a boosting coadditive alongside the commercial NIR laser marking additive (Iriotech 8815) in an epoxy system. Our findings demonstrate that the incorporation of Mg_2_Al-CO_3_ LDH can significantly increase both the darkness and contrast of marking even at high laser head speed (5000 mm/s), while minimizing surface damage. Notably, by replacing 95% of Iriotech 8815 with Mg_2_Al-CO_3_ LDH, an epoxy plate can exhibit high transparency, while producing dark, sharply defined markings with excellent readable QR code markings at high laser speeds. This result offers a promising solution for enhancing high-speed NIR laser marking on transparent polymers with additional advantages of lower toxicity and cost and with minimal optical interference from high additive loadings.

## Introduction

Surface labeling plays a crucial role across various industries, including manufacturing, healthcare, packaging, and consumer goods, providing comprehensive information like trademarks, compliance details, and product identification. However, the traditional labeling techniques, such as adhesive labels, painting, ink marking, and screen printing, have the problems of limited-durability, contamination, high cost, and environmental impacts. In particular, it significantly increases the difficulty of polymer product recycling. Laser marking is a promising method for surface labeling using a focused laser beam to irradiate materials, leading to highly precise, permanent, visible, and damage-free patterns on the substrate surface. This approach delivers excellent durability and is eco-friendly.^1^ Moreover, it is noncontact and does not require any media (e.g., ink), adhesive, or pretreatments. The generally accepted laser marking mechanism involves the material absorbing the laser energy upon surface irradiation, followed by the conversion of this energy into heat. This process can induce physical/chemical transformation (heating, melting, evaporation and decomposition), leading to surface effects via color change (e.g., carbonization), engraving (e.g., ablation/ejection), or foaming).^2−4^ There are several types of lasers with a wide wavelength range from ultraviolet (UV) to far-infrared (far-IR). The most commonly used lasers in the market are transversely excited atmospheric-pressure carbon dioxide laser (TEA CO_2_, 10640 nm), Nd:YAG laser (1064 nm, 532 nm, 355 nm), Nd:YVO_4_ laser (532 nm, 355 nm), and excimer lasers (XeCI at 308 nm, KrF at 248 nm, ArF at 193 nm).^3^ UV lasers with higher energy outputs have been widely used in different materials from metals, ceramics, wood to polymers. However, UV lasers often suffer from 1) frequent maintenance requirements (e.g., the need for frequent changes in the UV laser medium); 2) low power efficiency (around 0.2–2 %); 3) high energy consumption. In contrast, NIR lasers (e.g., Nd:YAG laser) are more attractive in the industry when considering sustainability and efficiency. They offer cost-effective maintenance, good operational lifespan, distinctive single signal, minimal background effects, and enhanced safety features. Unfortunately, most transparent polymers (such as polyethylene (PE), polypropylene (PP), thermoplastic polyurethane, and polyepoxides) do not have absorption at 1064 nm, resulting in no desirable surface marking after laser irradiation.^5,6^ Therefore, it requires a laser-sensitive additive that can absorb laser energy at 1064 nm and then transfer it to the polymer to produce high contrast marking.

The inorganic materials such as antimony-doped tin oxide (ATO),^7^ diantimony trioxide (Sb_2_O_3_),^6^ bismuth oxide (Bi_2_O_3_),^8^ bismuth oxychloride (BiOCl),^9^ Fe_3_O_4_/ZnO,^10^ molybdenum sulfide (MoS_2_),^11^ SiO_2_ waste,^12^ and modified montmorillonite^13^ have been reported as additives for NIR laser marking in polymers. It has been found that the laser marking performance can be improved by coating the substrate with a polymer that is more easily carbonized such as a polycarbonate, polystyrene, or polyimide.^5,14−16^ However, these efficient inorganic materials generally have problems. For example, they require a high loading to be effective, induce color and opaqueness, and are potentially toxic. Adding other polymers to the system also creates additional challenges related to end-of-life recycling. Carbon materials such as graphene and carbon nanotubes have also reported as additives for NIR laser marking due to their good photothermal conversion and extremely low density.^17−19^ However, those carbon materials also rely on a synergistic effect with the other polymers (such as polycarbonate or polystyrene). Furthermore, the introduction of carbon materials would introduce black coloration into these products, which may not be preferred in many applications. To date, the literature reports good laser marking using these additives normally requiring laser speeds around 450–1000 mm/s.^5,6,8,10−19^ Higher speed leads to poor marking with blurred edges. Achieving a high laser marking speed on transparent polymer substrates with high precision and resolution is still an unmet challenge for commercial production lines. Therefore, there is a demand to develop low toxicity, eco-friendly, colorless, laser marking additives that enable high contrast and fast laser marking speeds.

Layered double hydroxides (LDHs) are a class of 2D materials with a general formula  where M and M′ are typically divalent and trivalent metal cations octahedrally coordinated by OH groups, A*^n^*^–^ represents the charge-compensating intercalated anions.^20^ LDHs can be found in nature as hydrotalcite, Mg_6_Al_2_(OH)_14_(CO_3_)·4H_2_O. They can be readily synthesized, and commercial samples are available from companies such as Kisuma Chemicals B.V., Sasol Ltd., and Clariant AG. Besides, Mg*_x_*Al-based LDHs are generally considered to have good biocompatibility. Mg_3_Al-CO_3_ LDH has been approved by the U.S. Food and Drug Administration and has already been successfully commercialized as the antacid agent such as Talcid. In addition, the refractive index of Mg*_x_*Al-CO_3_ LDH (∼1.5)^21^ closely matches many polymeric materials, e.g., epoxy resins (∼1.50–1.59),^22^ PP (∼1.50–1.54),^23^ PE (∼1.52),^24^ allowing for the creation of highly transparent (low scattering) polymer/Mg_*x*_Al-CO_3_ LDH composites.^25,26^ Furthermore, the large bandgap for Mg*_x_*Al-CO_3_ LDH means it has no impact on the optical absorbance of the polymers. Although, Mg*_x_*Al-CO_3_ based LDHs has been widely used in many applications such as gas barrier,^27−29^ electrolysis,^30−32^ CO_2_ capture,^33−35^ catalysis,^36,37^ and biomedicine.^38^ We believe its use as a synergistic laser marking additive has not yet been explored. Mg_*x*_Al-CO_3_ based LDHs offer nontoxicity, cost-effectiveness, and minimal impact on optical properties and as such could be good candidates as laser marking coadditive in laser marking technologies.

Herein, we report the use of Mg_2_Al-CO_3_ LDH platelets to boost the effectiveness of a commercial laser marking additive (Iriotech 8815). The goal is to minimize the loading of Iriotech 8815 which poses toxicity concerns, while achieving good laser marking efficiency and fidelity at high laser scanning speed. Different ratios of Iriotech 8815 and Mg_2_Al-CO_3_ LDH were blended in a protype epoxy system. The transparency and laser marking response of these composites were evaluated by using UV–vis-NIR spectroscopy, scanning electron microscopy (SEM), and optical microscopy.

## Results and Discussion

**Figure 1:**
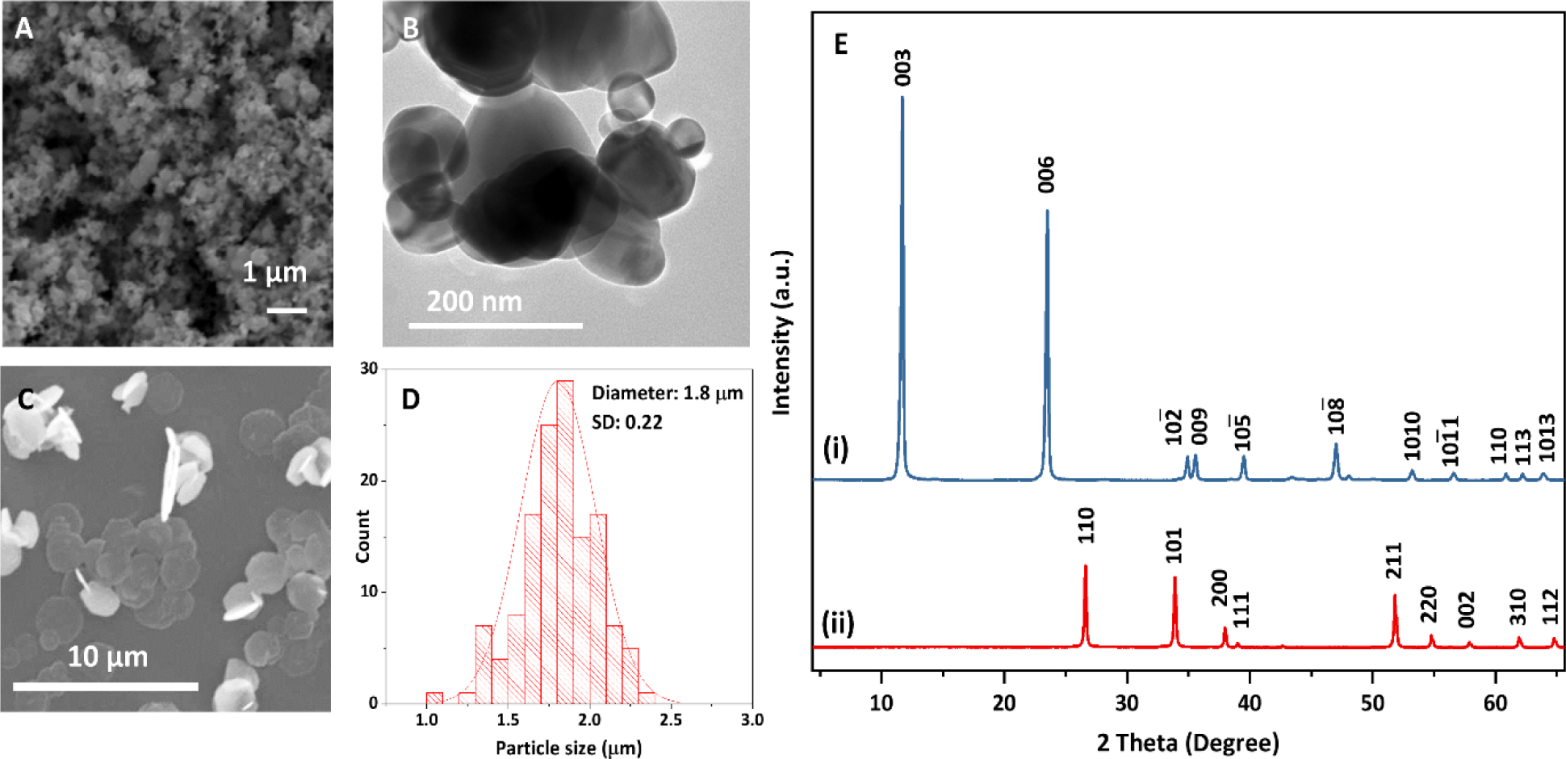
(A) SEM image and (B) TEM image of commercial laser additive (Iriotech 8815); (C) SEM image and (D) particle size distribution of Mg_2_Al-CO_3_ LDH platelets; (E) XRD patterns of (i) Mg_2_Al-CO_3_ LDH platelets and (ii) Iriotech 8815.

The commercial laser marking additive (Iriotech 8815) was sourced from Merck and used without further treatment. As shown in Figure 1A,B, Iriotech 8815 presents as small particles between 50–200 nm. The powder X-ray diffraction (XRD) in Figure 1E(ii) corresponds to tin oxide (SnO_2_, PDF#77–0477). The inductively coupled plasma mass spectrometry (ICP-MS) results indicate that the material also contains 0.18 wt % antimony doped in SnO_2_ crystalline. Mg_2_Al-CO_3_ LDH platelets were obtained using a urea method under hydrothermal treatment at 150 °C for 18 h. The synthesis details can be found in Supporting Information. As shown in the SEM image (Figure 1C), the obtained Mg_2_Al-CO_3_ LDH is monodispersed hexagonal platelets with a particle size of 1.8 ± 0.22 μm (Figure 1D).

**Figure 2:**
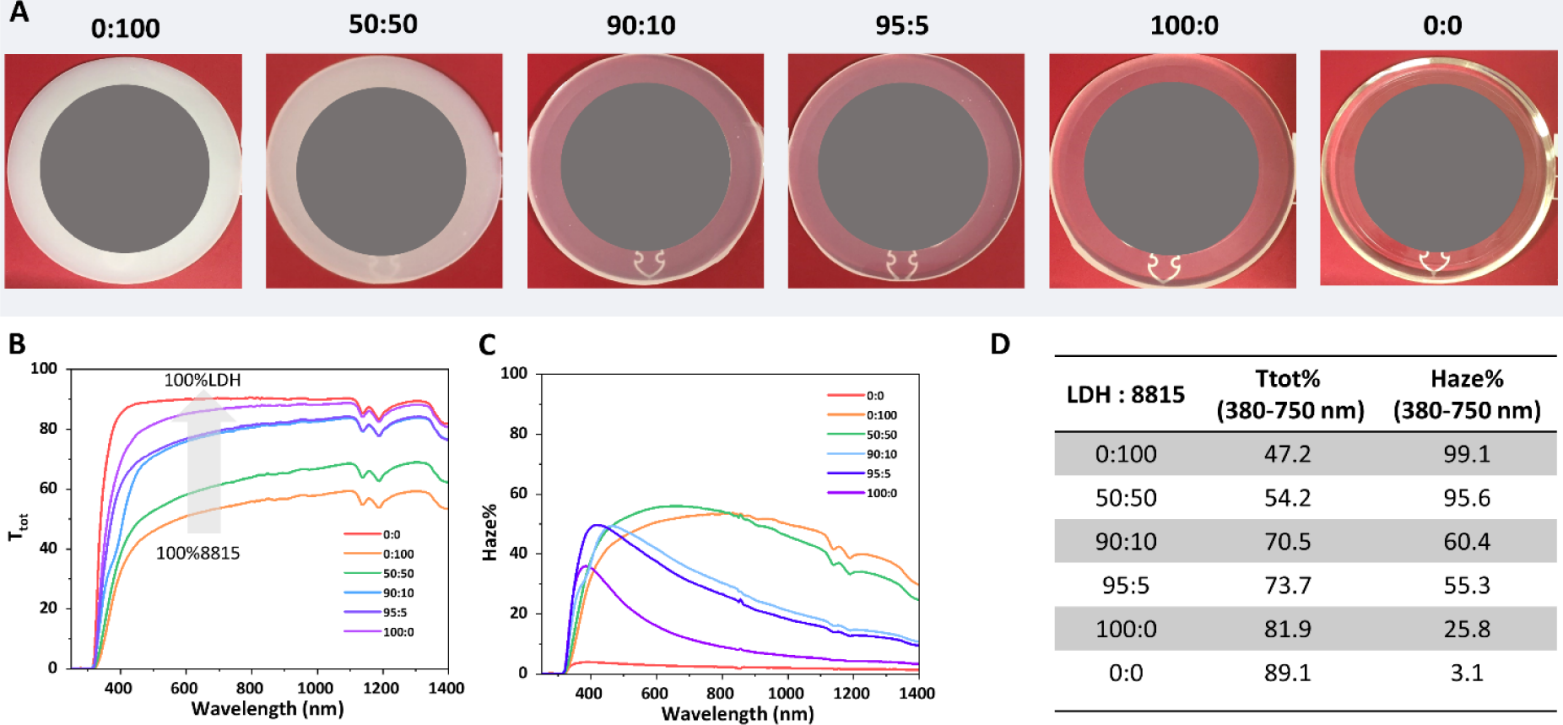
(A) Digital images of epoxy plates (read sheet is behind). (B) Total transmittance. (C) Haze (haze = diffuse transmittance/total transmittance x 100%). (D) Table of the average total transmittance and haze in the visible range for epoxy plates with different ratios of Mg_2_Al-CO_3_ LDH platelets and Iriotech 8815 (95:5 means 0.95 wt % Mg_2_Al-CO_3_ LDH platelets and 0.05 wt % Iriotech 8815, 0:0 means epoxy without any additive).

The powder XRD pattern indicates a high degree of order and regular stacking sequences within the LDH platelets. The Bragg reflections can be indexed to *R3̅m:H* rhombohedral symmetry lattice with unit cell parameters *a* = *b* = 3.04 Å, *c* = 22.74 Å. An epoxy plate without any additives (0:0) is highly transparent (Figure 2A), and the red sheet has been placed underneath as a visual guide to the optical transmission. The average total transmittance of the epoxy plate (Figure 2B) in the visible range (380–750 nm) is 89.1% with minimal haze of 3.1% (Figure 2C), where haze is the ratio of diffuse transmittance to total transmittance. After adding 1 wt % Iriotech 8815 (0:100), the sample becomes highly opaque. The total transmittance drops to 47.1%, and haze increases to 99.1%; this is not ideal in the marketplace. Addition of same amount (1 wt %) of our 1.8 μm Mg_2_Al-CO_3_ LDH platelet to the epoxy (100:0) results in an epoxy plate with high total transmittance (81.9%) and low haze (25.8%). We then systematically replaced Iriotech 8815 with Mg_2_Al-CO_3_ LDH while keeping the total additive content at 1 wt %. Samples with differing ratios Mg_2_Al-CO_3_ LDH:Iriotech 8815 (0:100, 50:50, 90:10, 95:05, and 100:0) were prepared. As shown in Figure 2, the plates became increasingly transparent as the LDH loading increased and the Iriotech8815 reduced. It is notable that the total transmittance remains relatively high (73.7%) when 95% of Iriotech 8815 was replaced with Mg_2_Al-CO_3_ LDH (Figure 2D).

**Figure 3:**
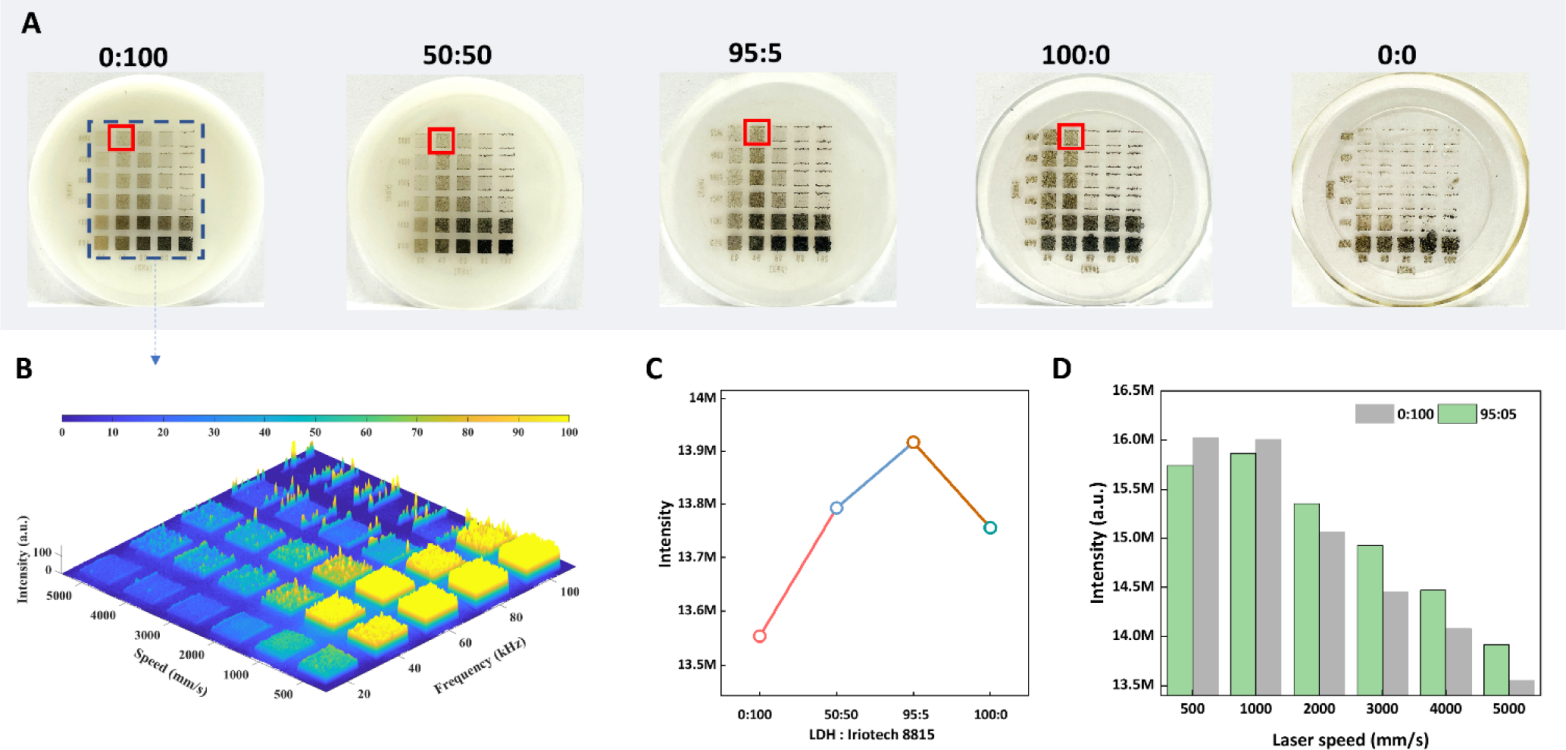
A) Digital photos of laser-marked epoxy plates containing 1 wt % additives with different ratios of LDH:Iriotech 8815 (laser wavelength:1065 ± 5 nm; the laser frequency 20–100 kHz from left to right, laser head speed 500–5000 mm/s from bottom to up). (B) 3D view of MATLAB image analysis of the 0:100 epoxy plate. (C) Intensity summary of region with pulse frequency at 40 kHz and laser head speed at 5000 mm/s. (D) Intensity comparison of epoxy plates (0:100) and (95:5) with various laser speed 500–5000 mm/s at pulse frequency of 40 kHz.

The laser marking process was conducted on these epoxy/composite plates using a range of laser pulse frequencies (20–100 kHz) and laser speeds (500–5000 mm/s). The conditions of the laser irradiation on the sample surface were set according to computerized vector coordinates as shown in Figure S1. The additive free epoxy plate (0:0) exhibits negligible marking (Figure 3A) when the laser speed exceeds 500 mm/s, as epoxy has minimal absorption at 1065 nm (Figure S2a). However, the epoxy does show black marking when a slow speed (500 mm/s) and high pulse frequency are used. The black coloration is a result of polymer carbonation due to the extremely high laser energy incident on the surface. As shown in Table S1, the laser energy reached on the sample surface increases with increasing frequency and/or decreasing laser head speed. At a slow speed (500 mm/s) and high frequency (100 kHz), the laser energy on the sample (2 x 2 mm) surface is up to 2 J. After incorporating 1 wt % Irotech 8815, the plate (0:100) exhibits black and smooth marking at low speed (500–1000 mm/s). This can be attributed to efficient energy absorption by Iriotech 8815 at 1065 nm (Figure S2) followed by local thermal energy transfer on the epoxy surface. The darkness of the marking is increased as pulse frequency increases due to the net higher laser energy input on the surface (Table S1). As the laser speed increases above 2000 mm/s, the markings become pale and at 5000 mm/s, it becomes barely visible as the laser energy on the surface is low (<0.1 J). When adding 1 wt % of Mg_2_Al-CO_3_ LDH which has no absorption at 1065 nm (Figure S2), it is surprising to discover that the epoxy plate (100:0) notably presents black markings over the entire speed range at low pulse frequency (20–40 kHz) or throughout the pulse frequency range at lower speeds (500–1000 mm/s). It is worth noting that even at high speed (5000 mm/s), distinct black markings are still observed. However, the marking is not uniform over the marking area. By blending the proportion of Mg_2_Al-CO_3_ LDH with the Iriotech 8815 at 1 wt % overall loading into the epoxy, it is found that all markings became darker in appearance compared to those made with pure Iriotech 8815. At high laser head speeds (e.g., 5000 mm/s) and frequency above 20 kHz, unusual markings were observed: none of the samples display marking at higher frequency (e.g., 100 kHz), despite the relatively higher laser energy than the ones at lower frequency (20 kHz). The reason for this phenomenon is still unclear.

To quantitatively assess the darkness of the markings, a MATLAB routine was developed to read the image and convert its RGB values into grayscale values. Figures 3B and S3A present the programmed 3D and 2D images of the epoxy plate (0:100), respectively, with the intensity indicating the darkness of the markings. It is evident that the markings exhibit significantly greater intensity in the low laser speed (500–1000 mm/s) and higher frequencies (40–100 kHz), while they have very weak intensity at 5000 mm/s speed, in agreement with the digital photos in Figure 3A. The MATLAB routine was applied for all other sample images of which 2D images are shown in Figures S3B-E. It is apparent that the plates blending Mg_2_Al-CO_3_ LDHs and Iriotech 8815 as coadditive exhibited notably higher intensities at high laser speeds. When plotting the intensity at 5000 mm/s and 40 kHz, it becomes clear that the intensity increases with increasing Mg_2_Al-CO_3_ LDHs loading and a decrease in the amount of Iriotech 8815. Surprisingly, the epoxy plate (95:5), in which 95% of Iriotech 8815 was replaced with Mg_2_Al-CO_3_ LDH, demonstrated the highest darkening intensity confirming a synergic boosting effect between Mg_2_Al-CO_3_ LDH and Iriotech 8815 when laser marking. This result not only means an enhancement of the marking darkness but also lowers the cost and minimizes toxicity. An example of detailed comparison at 40 kHz and a speed range of 500–5000 mm/s is presented in Figure 3D. At low laser speed (500–1000 mm/s), the plate (95:5) with Mg_2_Al-CO_3_ LDH/Iriotech 8815 ratio of 95:5 exhibits a lower marking intensity than that of the plate (0:100) with pure Iriotech 8815. While increasing laser speed up to 5000 mm/s, the marking intensity became much more intense compared to that of the plate (0:100), offering an attractive solution to high-speed production lines.

**Figure 4:**
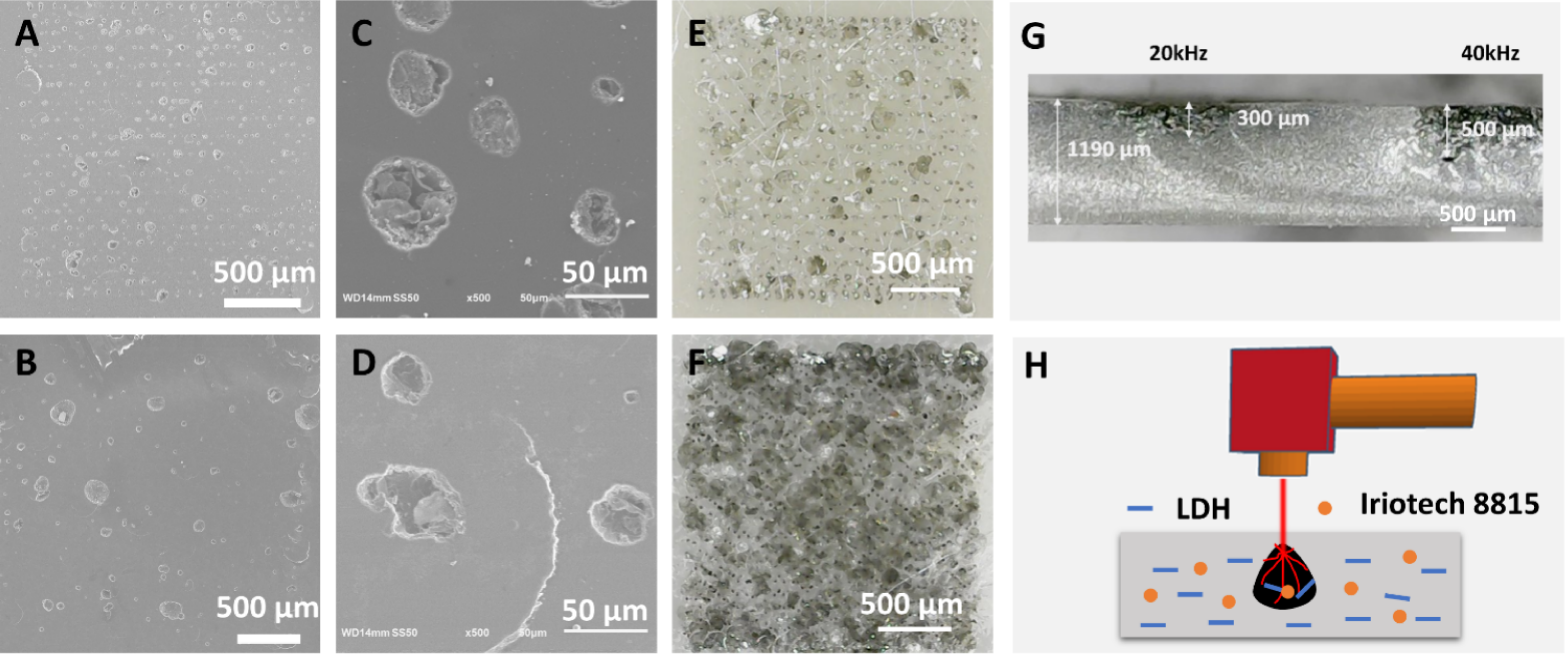
SEM images of laser-marked epoxy plates after laser marking (5000 mm/s and 40 kHz) (A) (0:100) and (B) (95:5) at low magnification; (C) (0:100) and (D) (95:5) at high SEM magnification. Optical microscopy images of laser-marked epoxy plates at 5000 mm/s and 40 kHz (E) (0:100) and (F) (95:5). (G) Cross-session optical image of laser-marked epoxy plate (95:5) at 500 mm/s and 20–40 kHz. (H) The schematic diagram of laser marking process.

The surface textures of the laser-marked plates were examined using SEM and optical microscopy (Figure 4A-F and Figures S4–S5). At a high speed of 5000 mm/s and a frequency of 40 kHz, laser irradiation on the epoxy plate (0:100) resulted in the formation of numerous surface holes within the laser marking region (Figure 4 A – C). Under optical microscopy, the markings present as a weak contrast but reflective around the holes due to high light scattering from the rough surface texture (Figure 4E). The surface of the epoxy plate (95:5) after laser irradiation under the same laser marking conditions presents much fewer surface holes (Figure 4B–D), but significantly blacker marking compared to that of the plate (0:100) (Figure 4F). Interestingly, the dark markings on the plates (95:5) observed from optical microscopy do not actually align with the pattern of the surface holes revealed from SEM. This suggests that the source of the black markings may originate from subsurface features rather than the light scattering caused by the surface holes. To further explore the source of the marking, a cross-session was analyzed using optical microscopy. It is found that the darkness extends into the plate with the length of approximately 300 and 500 μm after irradiation at 20 and 40 kHz, respectively (Figure 4G). As the laser frequency increases, the length of the darkened region increases, penetrating 900 μm at 500 mm/s and 100 kHz (Figure S5). These findings confirm that the black markings on the plate indeed originate beneath the surface layer (Figure 4H).

The influence of the laser speed on the surface was further investigated at 40 kHz (Figure S4A). Decreasing the laser speed resulted in varying degrees of damage to the surfaces of both the (0:100) and (95:5) plates, leading to a rough texture. Interestingly, under the same laser irradiation conditions, the plates (95:5) exhibit significantly less damage with fewer holes but notably darker markings compared to the plate (0:100). The formation of large surface holes in the plate (0:100) can be attributed to the highly efficient absorption of laser energy by Iriotech 8815 within the epoxy. Iriotech 8815 can instantly transfer this energy as heat, causing a rapid increase in the local temperature within the laser interaction region. This localized thermal shock and the rapid increase of the internal pressure results in a pyrolysis gas trigger and ejection/ablation of the material, leading to the formation of the surface holes.^39,40^ The large amount of heat is dispersed through material ablation and gas expulsion from the epoxy plates.^40^ This reduces the energy that is needed for thermal degradation of the remaining material, stopping the creation of black residues for laser marking. In contrast, the substitution of Iriotech 8815 by addition of Mg_2_Al-CO_3_ LDH in the epoxy may reduce rapid thermal fluctuations and overheating of the polymer, as evidenced by the reduction in the number of surface holes (Figure S4). The Mg_2_Al-CO_3_ LDH may offer an additional mechanism for energy dissipation beneath the surface, allowing more efficient thermal degradation of the epoxy and further carbonization. This process led to the formation of black residues below the surface. When the epoxy is blended with 0.05 wt % of Iriotech 8815 (equivalent to the Iriotech 8815 content in plate (95:5)), black markings are more apparent (Figure S6) compared to 1 wt % Iriotech 8815 content plate (0:100) (Figure 4E). However, its uniformity is diminished perhaps due to low thermal dissipation efficiency resulting from an extremely low concentration of Iriotech 8815 in the epoxy. In the (95:5) plate, the Mg_2_Al-CO_3_ LDH particles are well-dispersed in the epoxy system as evidenced by its high transparency (Figure 2A). This dispersion facilities the efficient distribution of thermal energy, leading to a controlled temperature rise that induces thermal degradation of epoxy material with the formation of black residues below the surface. Even without Iriotech 8815, the sample (100:0) displays black marking at high speed (Figure S7), suggesting that LDH may have the capability to absorb some laser energy and offer heat transfer to the epoxy. However, these markings exhibited much less clarity, probably due to poor energy absorption by the LDH. This results in a slower temperature rise, causing the epoxy to melt over a large area. The Raman spectra (Figure S8) revealed a new broad diffusion band in the range 1000–2000 cm^–1^ after laser marking. This is attributed to a mixture of disordered sp, sp^2^, sp^3^ carbon species,^41,42^ confirming that black residues are the amorphous carbon that likely result from the thermal decomposition/carbonization of epoxy.^6,14,16^

**Figure 5:**
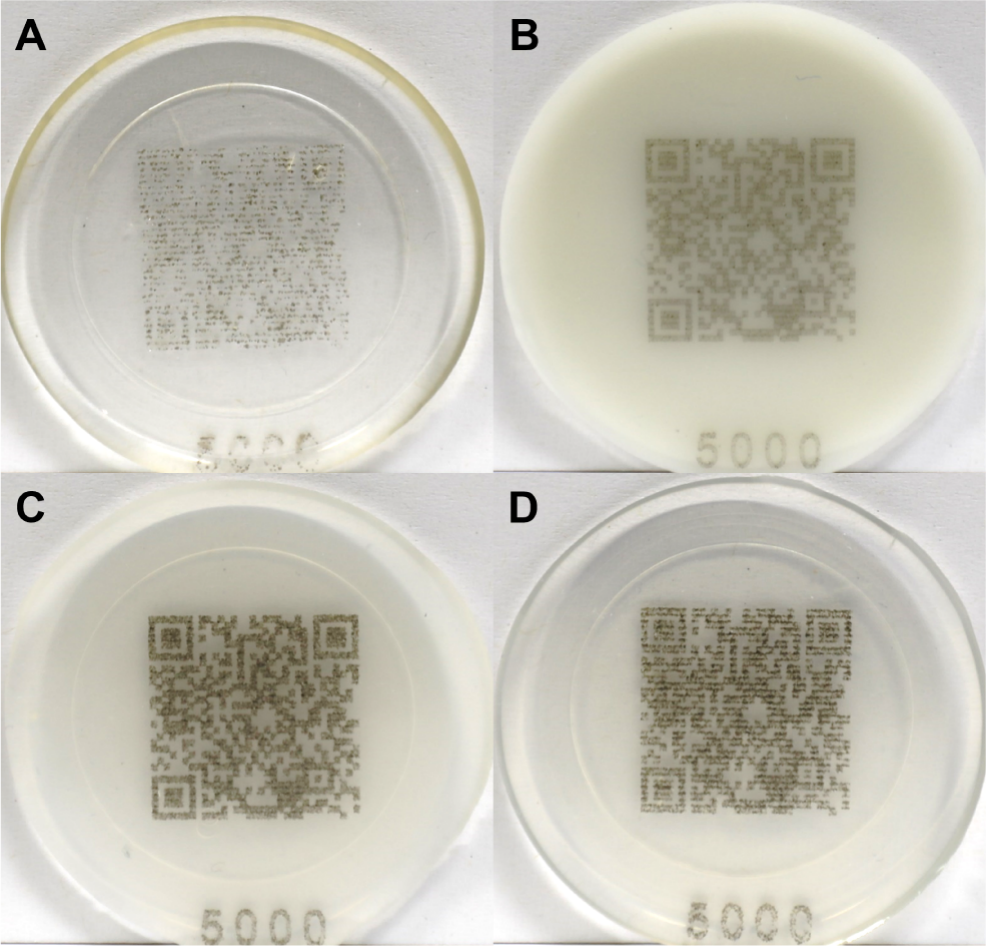
Digital photos of QR code-marked epoxy plates (A) 0:0, (B) 0:100, (C) 95:5, and (D) 100:0.

The image fidelity upon marking of the epoxy plates was evaluated by QR code marking at a fast laser head speed (5000 mm/s) and a laser pulse frequency of 40 kHz. As shown in Figure 5, the QR code on the pure epoxy plate exhibits a blurred QR code structure (Figure 5A), which is not detectable via a scanning device such as a smart phone. When blending 1 wt % of Iriotech 8815 into the epoxy, the QR code marking on the surface of the plate (0:100) is well-defined, with clear lines and edges, making it scannable with a smartphone (Figure 5B). However, the contrast between the marking and background is relatively low, resulting in a longer response read time after scanning. The incorporation of 1 wt % of Mg_2_Al-CO_3_ LDH into the epoxy results in a significantly darker QR code marking with enhanced contrast (Figure 5D). Nonetheless, the resolution and clarity of the markings are poor, leading to scanning difficulties. Remarkably, when epoxy is blended with 0.05 wt % Iriotech 8815 and 0.95 wt % Mg_2_Al-CO_3_ LDH, the plate (95:5) generates a clear QR code, with the black marking displaying excellent resolution and sharpness, allowing an instant response after scanning with a smartphone. These findings demonstrate a significant synergistic impact of the Mg_2_Al-CO_3_ LDH on Iriotech 8815 performance in the epoxy on the marking quality, with the plate (95:5) offering a particularly favorable incorporation of good darkness, high clarity, and resolution, facilitating rapid and accurate scanning.

The mechanical properties of the epoxy plates (0:0), (0:100) and (95:5) and their corresponding laser-marked plates (0:0 M), (0:100 M) and (95:5 M) were evaluated. ISO37 Type 3 dogbone specimens were prepared and measured with an Instron 5582 tensile tester. The results as shown in Figure S9 indicate that laser marking did not significantly affect the tensile strength of the epoxy plates. There was no significant decrease within the error in tensile strength when LDH/Iriotech 8815 (95:5) was added, suggesting that the LDH does not substantially compromise the mechanical properties of the epoxy.

## Conclusions

In summary, we have evaluated the impact of incorporating Mg_2_Al-CO_3_ LDH as a coadditive with Iriotech 8815 at varying ratios within an epoxy for NIR laser marking. The transparency and haze of the blended epoxy plates were analyzed using UV–vis-NIR with integrating sphere, the surface texture of the NIR laser markings on the plates was examined using SEM and optical microscopy, the quality of the markings was evaluated by a MATLAB routine and a QR code scanning response. Impressively, we found that the incorporation of Mg_2_Al-CO_3_ LDH (a non-NIR absorber) can significantly enhance the contrast of the NIR laser marking by producing black residues under the surface while retaining the relatively high transparency, low haze, and less surface damage with fewer holes. Of particular interest, the epoxy plate (95:5), replacing 95% of Iriotech 8815 with Mg_2_Al-CO_3_ LDH in epoxy, presents the markings that were both dark and sharply defined even at a high laser marking speed of 5000 mm/s. Its QR code marking exhibited excellent resolution and clarity, allowing for quick and accurate smartphone scanning. Our findings underscore the vital role of Mg_2_Al-CO_3_ LDH in enhancing NIR laser marking quality on non-NIR responsive polymer. This research sheds light on the potential for tailored epoxy/inorganic conposites that offer nontoxic, cost-effective, and minimal optical impact solutions while enhancing the NIR laser marking quality. This advancement creates opportunities for other transparent polymer systems such as PP (Figure S10), and PE. Recognizing the distinct physical and chemical properties, as well as laser response characteristics of different polymer materials, further efforts, particularly in refining the laser marking process and the polymer processing, are essential to tailor the method to each specific material to achieve optimal performance.

## Experimental Section

### Mg_2_Al-CO_3_ LDH Platelet Synthesis

A mixture of Mg(NO_3_)_2_.6H_2_O (10 mmol), Al(NO_3_)_3_.9H_2_O (5 mmol), and urea (30 mmol) was dissolved in 500 mL of deionized (DI) water. The solution was hydrothermally treated at 150 °C for 18 h in Parr reactor 4520. The solid was collected and washed with DI water until pH7 followed by ethanol (635 mL). The solid was then dispersed into ethanol (625 mL). After 1 h, the solid was filtrated and washed with ethanol (625 mL) before it was dried in vacuum oven.

### Epoxy Composition Preparation

The Araldite DBF (1.25 mL) was mixed with sample powder (1 wt % of epoxy mass (Araldite DBF + Aradur HY 2966)) using a high speed mixer without vacuum at increased speeds from 500 rpm for 1 min, 1000 rpm for 1 min, and finally 2000 rpm for 1 min. HY 2966 (0.375 mL) was then added into the suspension and mixed using a high speed mixer at increased speeds and vacuum: 500 rpm for 1 min without vacuum, 1000 rpm for 1 min under 30 kPa and then 2000 rpm for 2 min under 5 kPa vacuum. The sample was then placed in a vacuum oven at 60 °C overnight. The ratio of LDH to Iriotech 8815 is varied from 0:100, 50:50, 90:10, 95:05 to 100:0.

### Laser Marking

The laser marking was conducted by using a diode-pumped, q-switched fiber laser (Rofin Powerline F20) with laser wavelength of 1065 ± 5 nm, and laser power was fixed at 20 W for all samples. The sample was placed at the center of the sample stage. The laser irradiates on the sample following the vector pattern with coordinates of frequency and speed (Figure S1). The coordinate at the horizontal axis represents the laser pulse frequency (20–100 kHz), while the one at the vertical axis is the laser head scanning speed (500 to 5000 mm/s). The QR code was marked at a high speed of 5000 mm/s and pulse frequency of 40 kHz.

## Supporting Information Available

Materials, characterizations, computerized vector image with coordinates, 2D MATLAB-scripted images, UV–vis-NIR absorption, SEM, and optical microscopy images (PDF)
