# Supplementary material for: Boosting NIR Laser Marking Efficiency of a Transparent Epoxy Using a Layered Double Hydroxide
Source: ACS Appl Polym Mater. 2024 Jul 5;6(14):8679–86. doi: 10.1021/acsapm.4c01815 (PMC11287490; doi:10.1021/acsapm.4c01815)
Supplement: Supplementary file 1 — ap4c01815_si_001.pdf [file ap4c01815_si_001.pdf]

Supporting Information

Boosting NIR Laser Marking Efficiency of a Transparent Epoxy using a Layered Double Hydroxide

Chunping Chen*, Junxin Wang†, Alexander Evans and Dermot O'Hare*

Chemistry Research Laboratory, Department of Chemistry, University of Oxford, 12 Mansfield Road, Oxford, OX1 3TA, UK

.

Corresponding authors:

E-mail: chunping.chen@chem.ox.ac.uk (Chunping Chen); dermot.ohare@chem.ox.ac.uk

(Dermot O'Hare)

1 General details

1.1 Materials

Magnesium nitrate hexahydrate ($Mg(NO_3)_2{\cdot}6H_2O$ (AR)), aluminium nitrate nonahydrate ($Al(NO_3)_3{\cdot}9H_2O$ (AR), Urea (AR), ethanol were purchased from Sigma-Aldrich Co. LLC and used without further purification. Deionised water (DI) was used throughout the experimental process. Iriotech 8815 was obtained from Merck and used without further purification. The Araldite DBF and Aradur HY 2966 were from Huntsman and used without further purification.

1.2 Characterisation

Powder X-ray Diffraction (XRD) XRD data were collected on a PANAnalytical X'Pert Pro diffractometer in reflection mode at 40 kV and 40 mA using Cu Kα radiation (α1 = 1.54060 Å, α2 = 1.54426 Å, weighted average = 1.54178 Å). Scans were recorded from 5° ≤ 2θ ≤ 70°. Samples were mounted on stainless steel sample holders; peaks produced from these holders are observed at approximately 43 and 50°.

Inductively Coupled Plasma Mass Spectrometry (ICP-MS) ICP-MS analysis was performed by Dr. Alaa Abdul-Sada at the University of Sussex on an Agilent 7500 Series ICP-MS in helium collision mode. Approximately 30 mg of the sample was dissolved in 10 mL of 10% nitric acid solution. The solutions were then diluted by a factor of 100 with dilute nitric acid prior to analysis. Three repeats were performed for each measurement and the average was recorded.

Transmission Electron Microscopy (TEM) All TEM images were obtained on a JEOL 2100 microscope with an accelerating voltage of 200 kV. Samples were prepared by

dispersing particles in water or ethanol via sonication for 1 hour before casting onto carbon-coated copper grids.

Scanning Electron Microscopy (SEM) All SEM images were obtained on a JEOL JSM 6010LV scanning electron microscope with an accelerating voltage of 15 kV. The powder samples were dispersed in ethanol and then dried on silicon wafer while the epoxy plates were placed directly on the sample holder using carbon tape. All samples were coated with platinum using a Quorum SC 7620 Sputter Coater for 60 s before imaging.

Optical Microscopy All images were obtained on USB Bysameyee Digital Microscope using 8 LED mini video camera.

Optical measurement the optical measurements (total transmittance, diffuse transmittance and absorption) were conducted by using the UV-Vis-NIR spectrophotometer (PerkinElmer Lambda 1050+) with a 150 mm diameter integrating sphere.

Raman Spectroscopy the Raman spectra were collected from epoxy plates using a DXR3 Smart Raman Spectrometer with a 785 nm excitation laser.

Tensile Strength Samples were prepared via high-speed mixing under vacuum and cast in a silicon mold - ISO 37 Type 3 dogbone (width b_1 = 4 mm, length L_0 = 10 mm, thickness *ca*. 4 mm). Before measurements, samples were conditioned for 7 days at room temperature. The measurements were performed using an Instron 5582 tensile tester equipped with a 5 kN load cell. A grip-to-grip separation of 40 mm was used. The samples were pre-stressed to 3 N, then loaded with a constant cross-head speed of 100 mm/min. To calculate the tensile strength as stress (MPa), the reported force value was divided by the cross-sectional area (*ca.* 20 mm^2) of the specimens. The reported values are an average of at least 5 measurements of each composition and reported error ± 1σ.

2 Supporting Figures

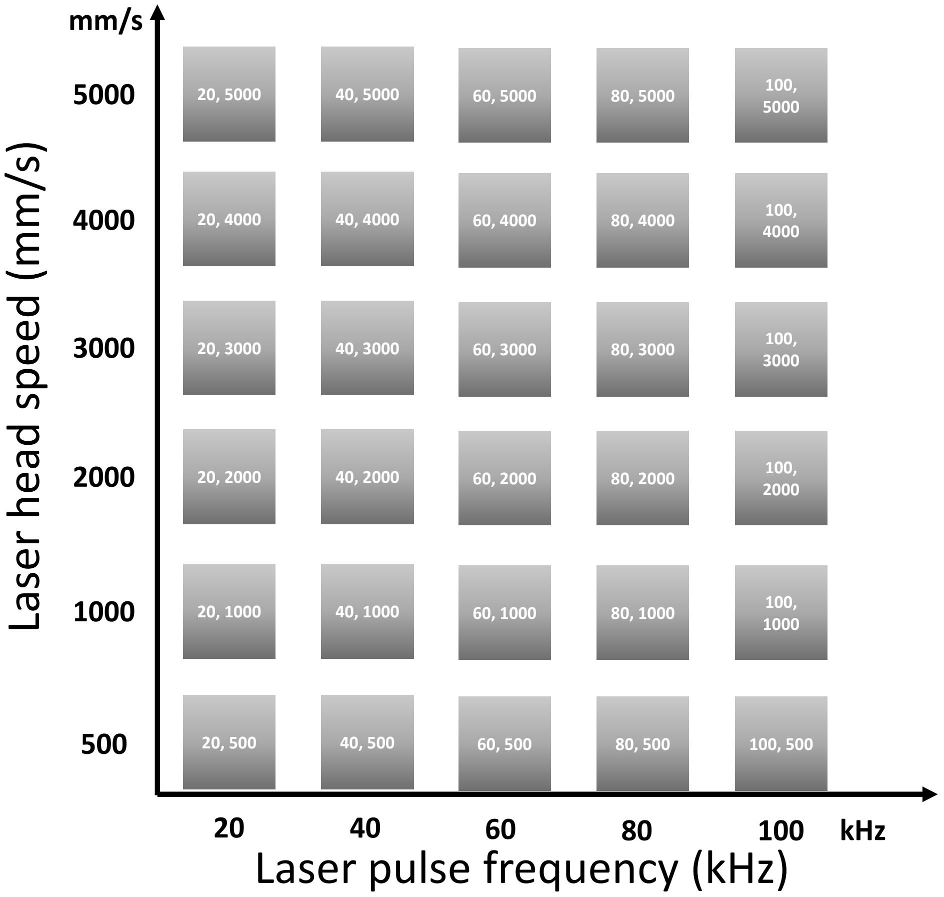

Fig. S1. The computerised vector image with coordinates (frequency, Speed)

Table S1 the laser energy (J) on the samples

Frequency (kHz) / Speed (mm/s)	20	40	60	80	100
**5000**	0.04	0.08	0.12	0.16	0.20
**4000**	0.05	0.10	0.15	0.20	0.25
**3000**	0.07	0.13	0.20	0.27	0.33
**2000**	0.10	0.20	0.30	0.40	0.50
**1000**	0.20	0.40	0.60	0.80	1.00
**500**	0.40	0.80	1.20	1.60	2.00

The calculation is shown here step by step, take a sample square (5000 mm/s and 100 kHz) as example:

Power is 20 Watt, Pulse width is 5 µs, spot size of the lens is 40 µm, sample square is 2 mm x 2 mm, the line width is 0.04 mm, frequency is 100 kHz and the speed is 5000 mm s^{-1}.

Laser energy per pulse (J): $20\, J.s^{-1} \times 5 \times 10^{-6} s = 1 \times 10^{-4} J$

The time for lase to scan one line in the square (s): $\frac{2\, mm}{5000\, mm/s} = 4 \times 10^{-4}\, s$

The number of pulses for one line: $4 \times 10^{-4}\, s \times 100 \times 10^{3} = 40$

The energy in one line (J): $40 \times 1 \times 10^{-4}\, J = 4 \times 10^{-3} J$

The number of lines in the square: $\frac{2\, mm}{0.04\, mm} = 50$

The energy in the square (J): $4 \times 10^{-3} J \times 50 = 0.2\, J$

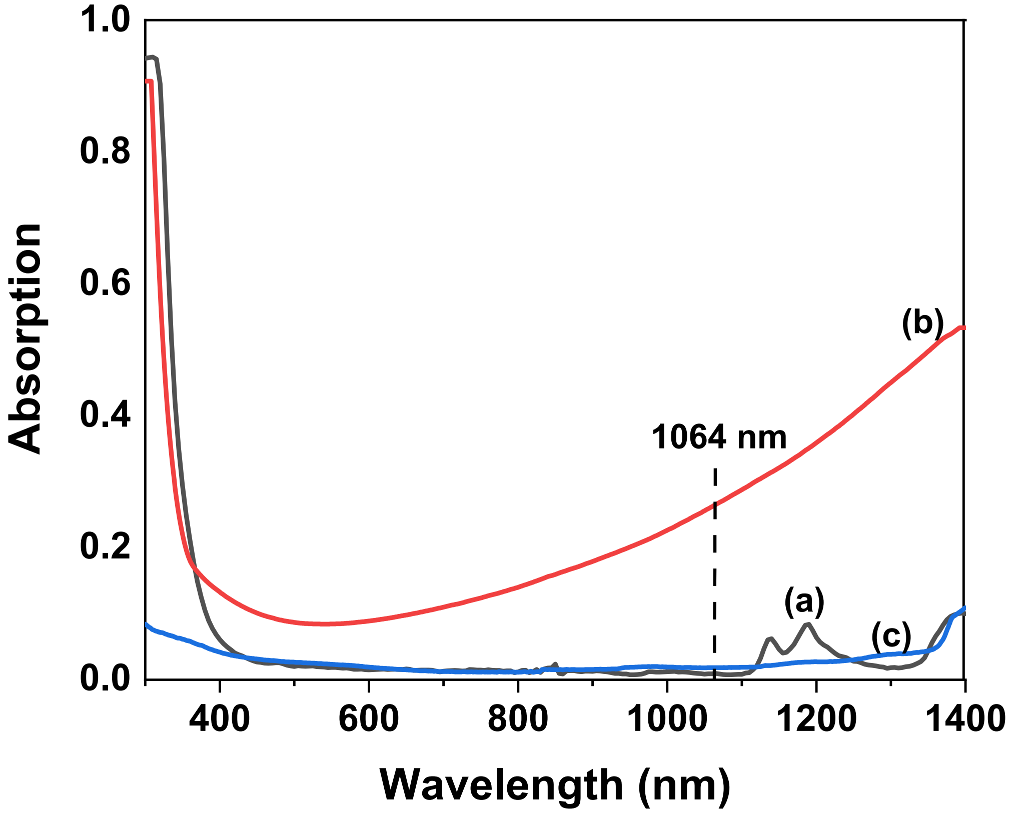

Fig. S2. UV-vis-NIR absorption of (a) pure epoxy, (b) Iriotech 8815 and (c) $Mg_2Al\text{-}CO_3$ LDH.

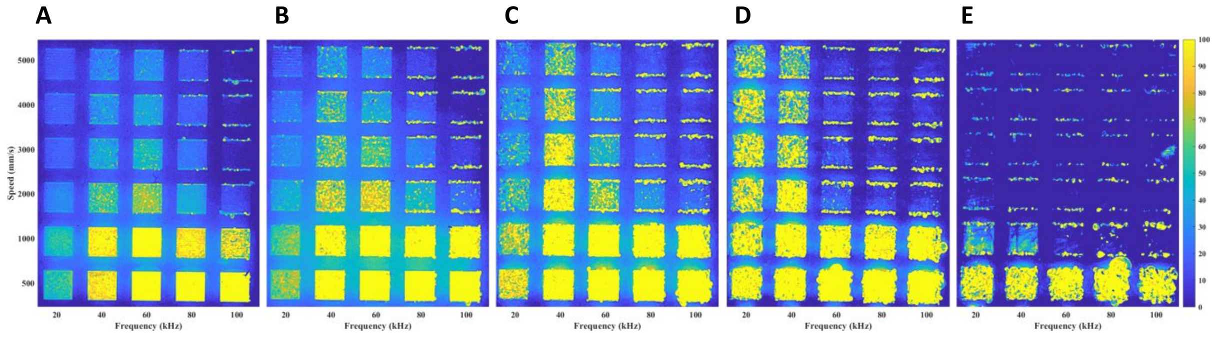

Fig. S3. 2D programmed images of epoxy plates with different $Mg_3Al\text{-}CO_3$ LDH: Iriotech 8815 (A) 0:100, (B) 50:50, (C) 95:5, (D) 100:0 and (E) 0:0.

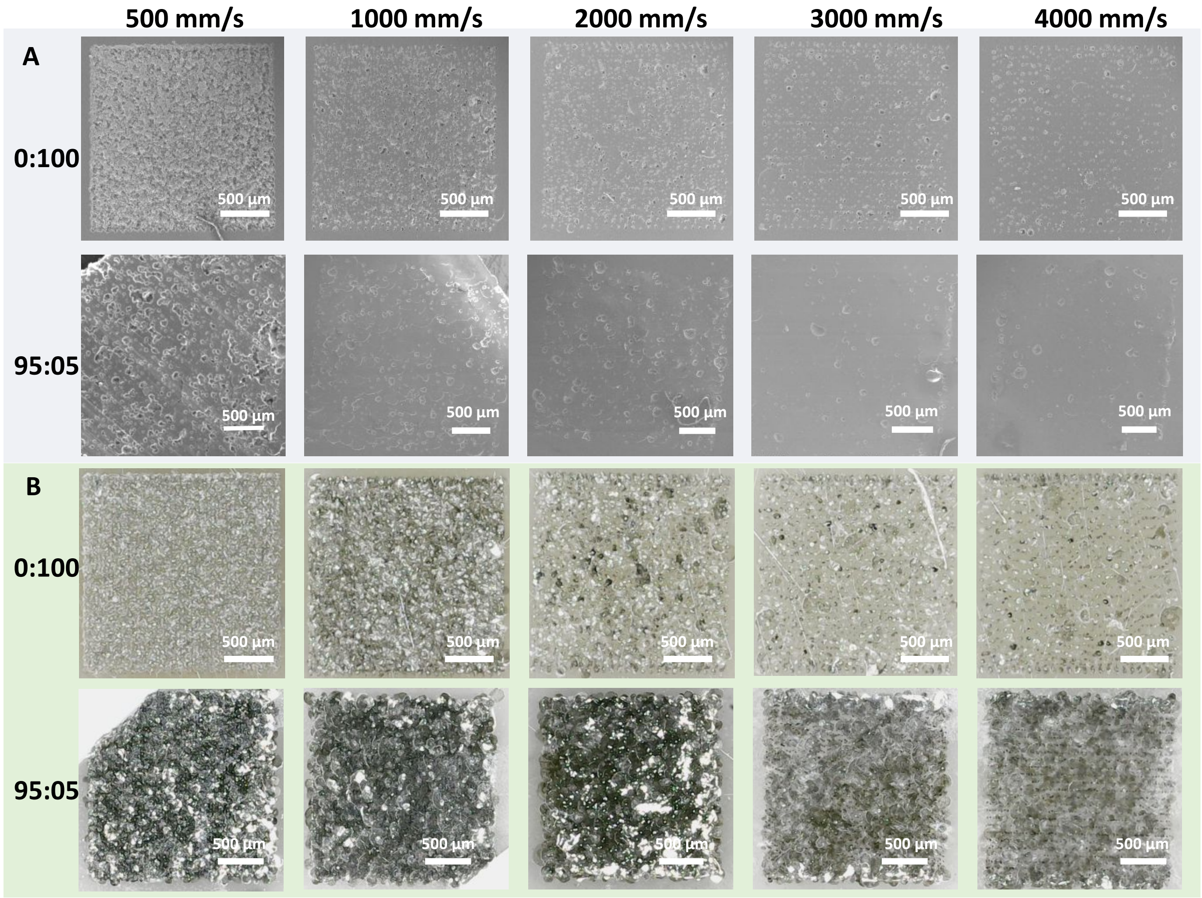

Fig. S4 (A) SEM images and (B) Optical microscopy images of epoxy plates (0:100 and 95:05) lasered marked at 40 kHz and laser head speed varied from 500 – 5000 mm.

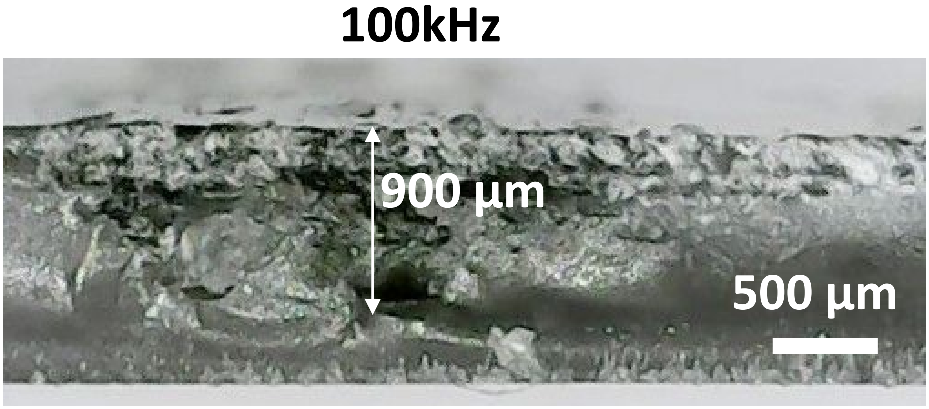

Fig. S5 Optical microscopy image of cross-sections of epoxy plate (100:0) lasered marked at 500 mm/s speed and 100 kHz.

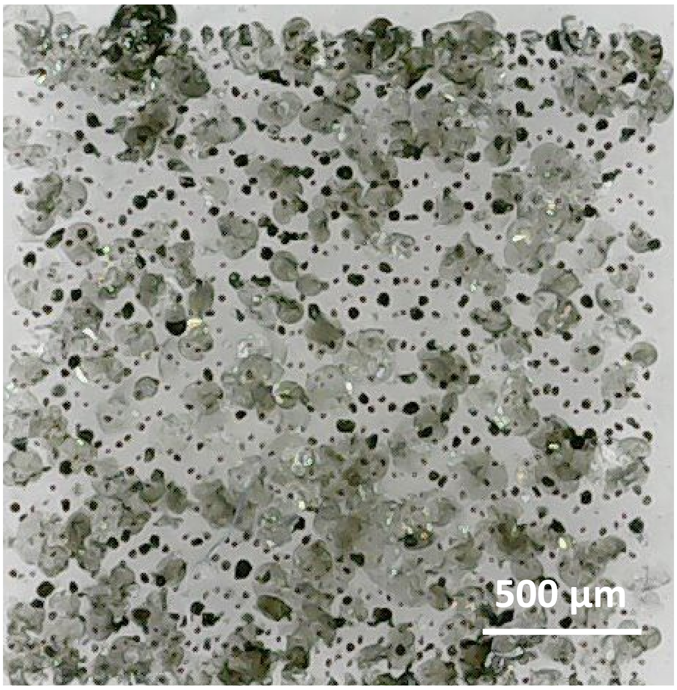

Fig. S6 Optical image of laser marked epoxy plates blended with 0.05wt% Iriotech 8815 at 5000 mm/s and 40 kHz.

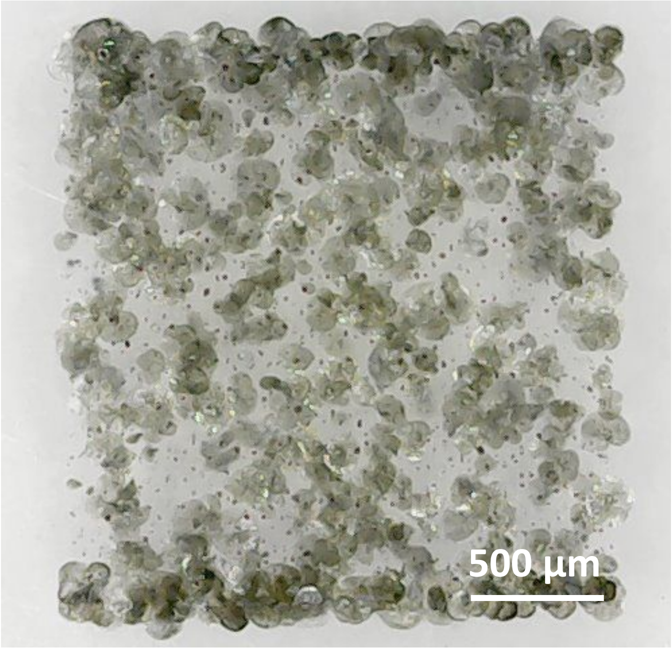

Fig. S7 Optical image of laser marked epoxy plates (100:0) at 5000 mm/s and 40 kHz.

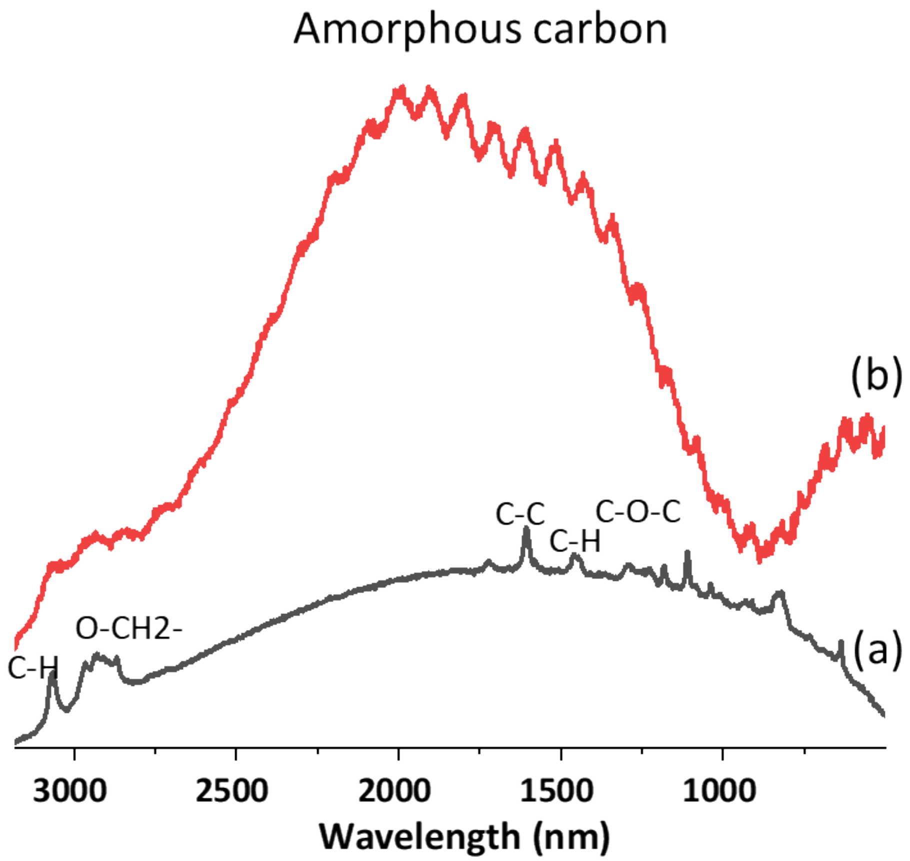

Fig. S8 Raman spectra of (a) epoxy plate and (b) laser marked on epoxy plate (100 kHz, 500 mm/s)

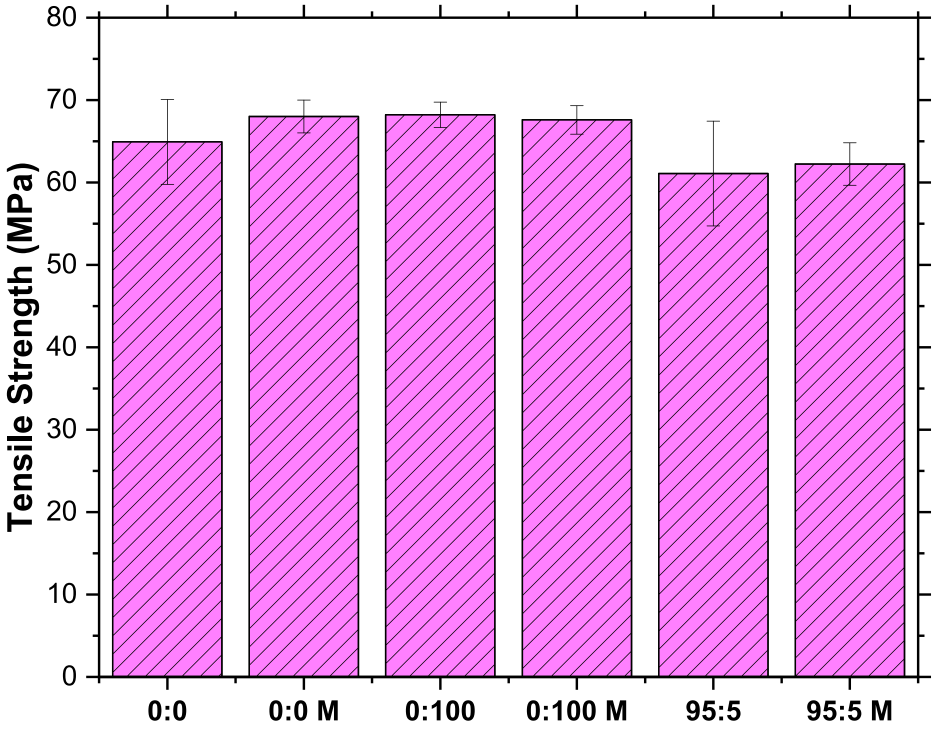

Fig. S9 Tensile strength of epoxy plates (0:0), (0:100), (95:5) and their laser marked plates ((0:0 M), (0:100 M) and (95:5 M) at 40 kHz, 5000 mm/s.

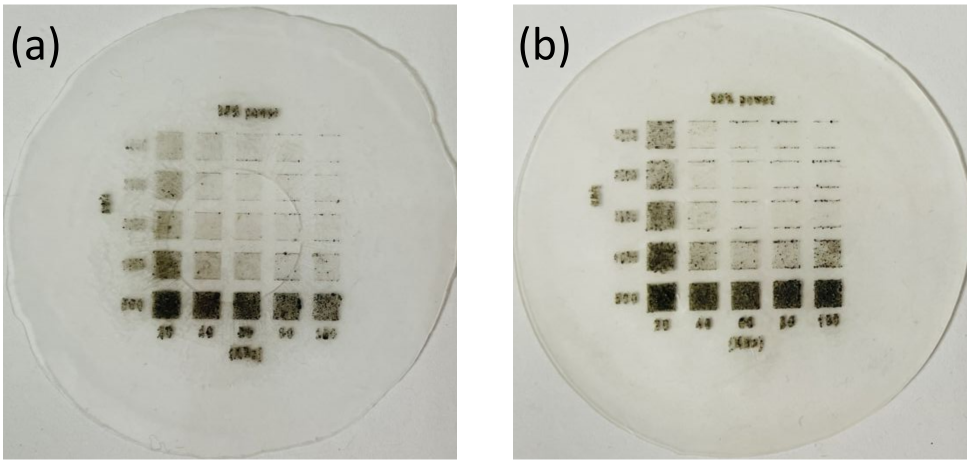

Fig. S10 Digital photos of laser marked polypropylene film (a) (0:0) and (b) (95:05), laser wavelength:1065 ± 5 nm; the laser frequency 20 – 100 kHz from left to right, laser head speed 500 – 5000 mm/s from bottom to up.
